# Latanoprost Acid–Brimonidine, a New Amide Prodrug for Glaucoma Management Based on the Concept of Sustained Release

**DOI:** 10.3390/molecules31162780

**Published:** 2026-08-10

**Authors:** Hong-Jia Lin, Shih-Horng Su, Wen-Chung Wu

**Affiliations:** 1 Department of Chemical Engineering, National Cheng Kung University, Tainan 701, Taiwan; 2 DuNing Incorporated, Tustin, CA 92780, USA

**Keywords:** glaucoma, latanoprost, brimonidine, prodrug, sustained release

## Abstract

Glaucoma is an ocular disease caused by the improper management of elevated intraocular pressure (IOP). IOP-lowering via topical administration is the first choice to prevent further progression. However, patients may forget to administer their medication, compromising IOP control. To address this problem, a prolonged active pharmaceutical ingredient (API) release system is proposed. A new prodrug (latanoprost acid–brimonidine conjugate, LBJ) was designed and expected to achieve potential long-lasting release of APIs. LBJ was synthesized by two methods. First, Steglich esterification without protecting the hydroxyl group led to a yield of 34.24%. However, the integral ratio between LPA and BM obtained from NMR was 1.25:1, indicating a potential side product resulting from further coupling through the unprotected hydroxyl group in LBJ. As an alternative route, Steglich esterification with a protecting agent, tert-butyldimethylchlorosilane (TBDMSCl), resulted in a yield of 39.35%, and the integral ratio between LPA and BM obtained from NMR was 1:1. The hydrolysis time of LBJ was investigated and compared with that of latanoprost (LP). In the presence of esterase (0.4 U/mL), the hydrolysis times of LP and LBJ were 4 h and 28 days, respectively. The prolonged hydrolysis time results in sustained APIs release, which is beneficial for the development of a sustained drug release system.

## 1. Introduction

Improperly managed IOP carries a high risk of progressive, irreversible vision loss and ultimately blindness. In a normal population with an IOP between 11 and 21 mmHg, the secretion and drainage of aqueous humor, starting from the ciliary body, moving through the posterior and anterior chambers, and then reaching the trabecular meshwork or uveoscleral pathway, remain balanced. However, in glaucoma patients, abnormalities occur in the aqueous humor drainage pathway, disrupting this balance and leading to elevated IOP [1,2].

Current strategies for glaucoma management include preventing further elevation in IOP. The first-line approach in IOP management is topical administration due to its non-invasive nature and ease of use [3,4]. However, low bioavailability is the inherent limitation of topical administration, and periodic administration is required. Generally, after topical administration, the drugs pass through the tear film and then reach the cornea surface. In this process, approximately 80–90% of the drugs are removed from the ocular surface due to the rapid turnover of tears and nasolacrimal drainage [5]. After that, the cornea’s complex structure forms another physical barrier to drug penetration. Five different layers form the cornea: from the outermost to innermost, the epithelium, Bowman’s membrane, the stroma, Descemet’s membrane, and the endothelium [6,7,8,9]. The hydrophobic properties of the epithelium form the first barrier to hydrophilic drugs, preventing them from penetrating more deeply [5,8,9]. However, the stroma, which makes up 90% of the cornea, is hydrophilic [5,9]. Thus, the balance between hydrophobicity and hydrophilicity is a crucial issue in drug design. Ultimately, approximately 1% or less of applied drugs are absorbed into the eye [5,8]. Consequently, attentional administration of eye drops becomes essential for patients to self-manage IOP. 

Prodrugs are medically inactive compounds that require enzymatic or chemical conversion to become active pharmaceutical ingredients (API). This conversion could be achieved by breaking the chemical bond, usually an ester or amide, between a parent drug (API) and a promoiety. In eye drops, prodrugs are commonly used to improve ocular absorption, residence time, and corneal penetration. In most cases, prodrugs are designed to increase the lipophilicity (compared to their parent drug) to improve the permeation through the corneal epithelium layer. Then, esterases or peptidases cleave the promoiety and release the API [8,9,10].

**Figure 1:**
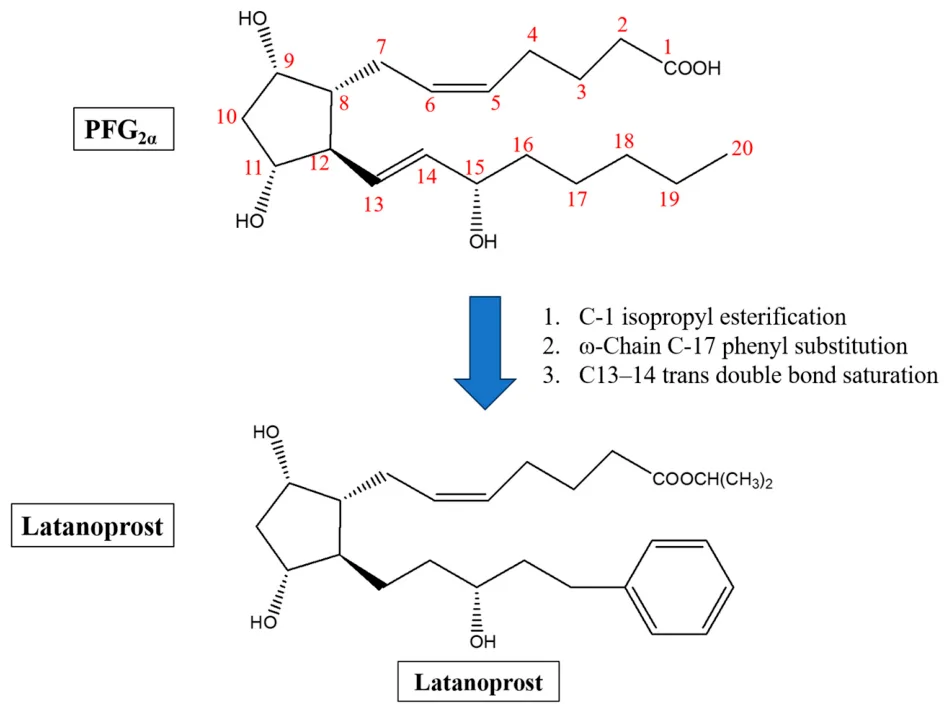
The development of Latanoprost.

Latanoprost is an isopropyl ester prodrug of a prostaglandin F_2α_ (PGF_2α_) analogue developed through the collaboration between Columbia University and Pharmacia (the pharmaceutical company) [11]. In the 1930s, prostaglandins were first isolated from prostate tissue [12], and in the 1980s, Bito discovered their ability to reduce IOP by increasing aqueous humor outflow through the uveoscleral pathway [13]. Initially, esterification was introduced to modify the PGF_2α_ to improve bioavailability, and isopropyl ester showed the best performance. Later, to reduce the adverse events, the researcher modified the length of the ω-chain and replaced the C-17 position with a phenyl group. Furthermore, they saturated the C13-14-trans double bond to improve the chemical stability. Figure 1 shows the structure of PGF_2α_ and latanoprost [11,14].

After administration, LP passes through the cornea and is rapidly hydrolyzed to the LPA by esterases. As a result, the LPA binds to the prostaglandin FP receptors distributed in ocular tissues, such as the ciliary epithelium and ciliary muscle, and upregulates the expression and promotes the release of matrix metalloproteinases (MMPs) [15,16]. These MMPs contribute to the extracellular matrix (ECM) remodeling [17,18], leading to expansion of tissue spaces within the uveoscleral pathway, thereby enhancing aqueous humor outflow and reducing IOP [19,20,21].

Brimonidine is another anti-glaucoma medication that belongs to the class of selective alpha2-adrenergic receptor agonists. Its hypotensive mechanism involves decreasing aqueous humor production and increasing uveoscleral outflow. In animal models, BM shows neuroprotective effects via promoting retinal ganglion cell survival and maintaining physiological function [22,23,24]. Such a mechanism enables BM to serve as an adjunctive therapy to LP, further enhancing the IOP-lowering effect. Research conducted by David A. Lee’s team showed that the mean additional IOP reduction was 5.89 mmHg when BM was used as an adjunctive therapy to LP [25]. Similar conclusions were also drawn by other researchers [26].

**Scheme 1:**
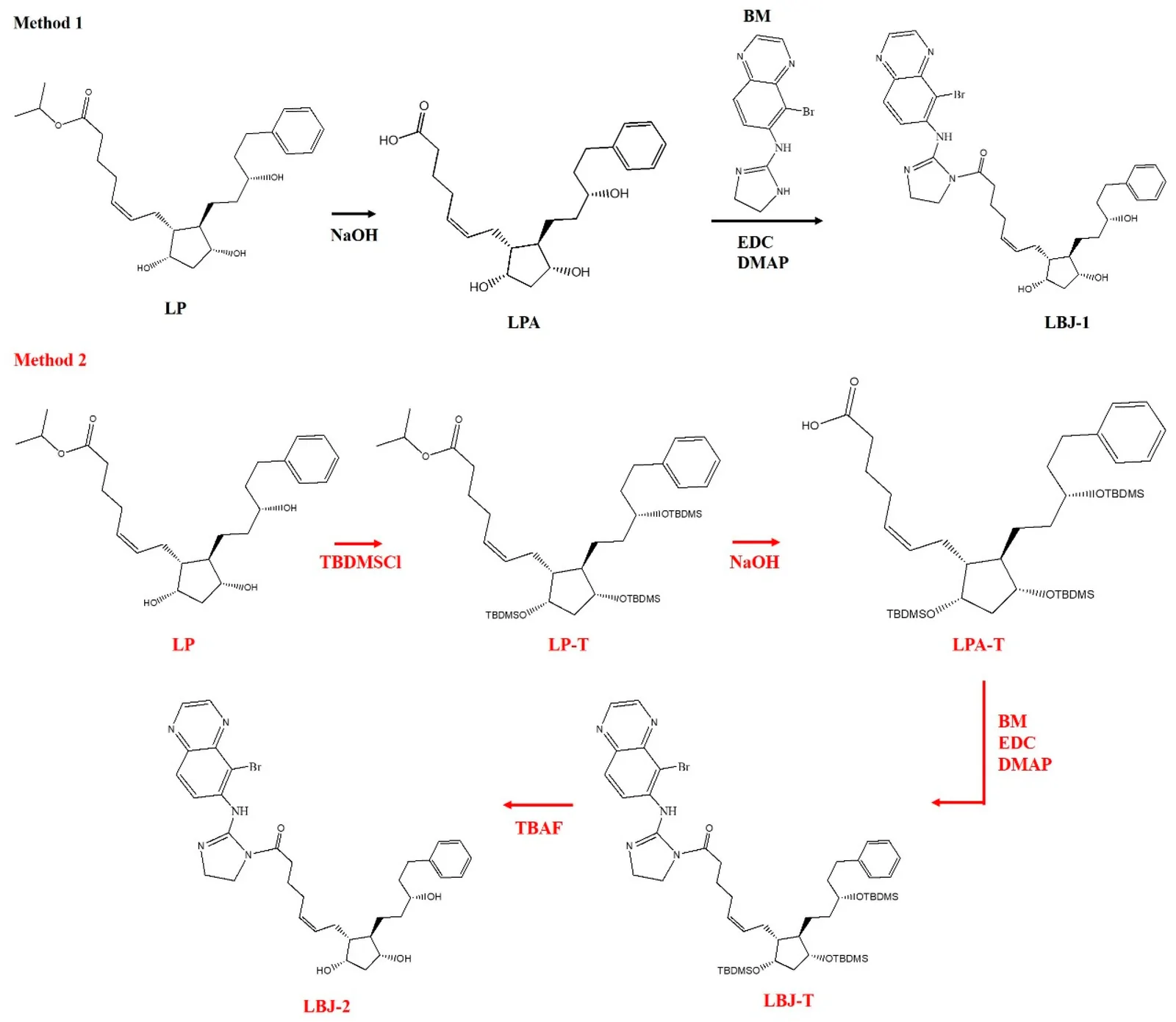
The synthesis of LBJ.

The daily use of a combination of latanoprost and brimonidine at fixed concentrations increases the bioavailability of glaucoma drugs and effectively lowers IOP. However, patients’ forgetfulness remains to be addressed. The purpose of this study is to create a new prodrug by using BM as the promoiety of LPA. The synthetic pathway of LBJ is shown in Scheme 1. This latanoprost-brimonidine conjugate is hypothesized to be hydrolyzed more slowly, resulting in a long-lasting release of the API.

## 2. Results and Discussion

### 2.1. Synthesis of LBJ

To investigate the hypothesis that extending the hydrolysis time of a prodrug could provide sustained release, LBJ, an amide prodrug consisting of LPA and BM, was synthesized in this study. Two methods were used to synthesize LBJ, including Steglich esterification with or without the use of the protecting agent (TBDMSCl).

#### 2.1.1. Synthesis and Characterization of LBJ Through Method 1 (LBJ-1)

Steglich esterification involving the coupling agent (EDC) and DMAP is commonly used to synthesize ester compounds under moderate conditions and can also be used to form an amide bond [27]. LP was initially hydrolyzed into LPA under basic conditions. Subsequently, LPA and EDC were reacted at room temperature with moderate stirring to form the *O*-acylurea intermediate. Without the presence of DMAP, O-acylurea intermediate may slowly transfer to an irreversible urea side product. Finally, the nucleophilic amine group at the BM attacked the acyl group of the intermediate to form the product LBJ-1, as a yellow solid type.

**Figure 2:**
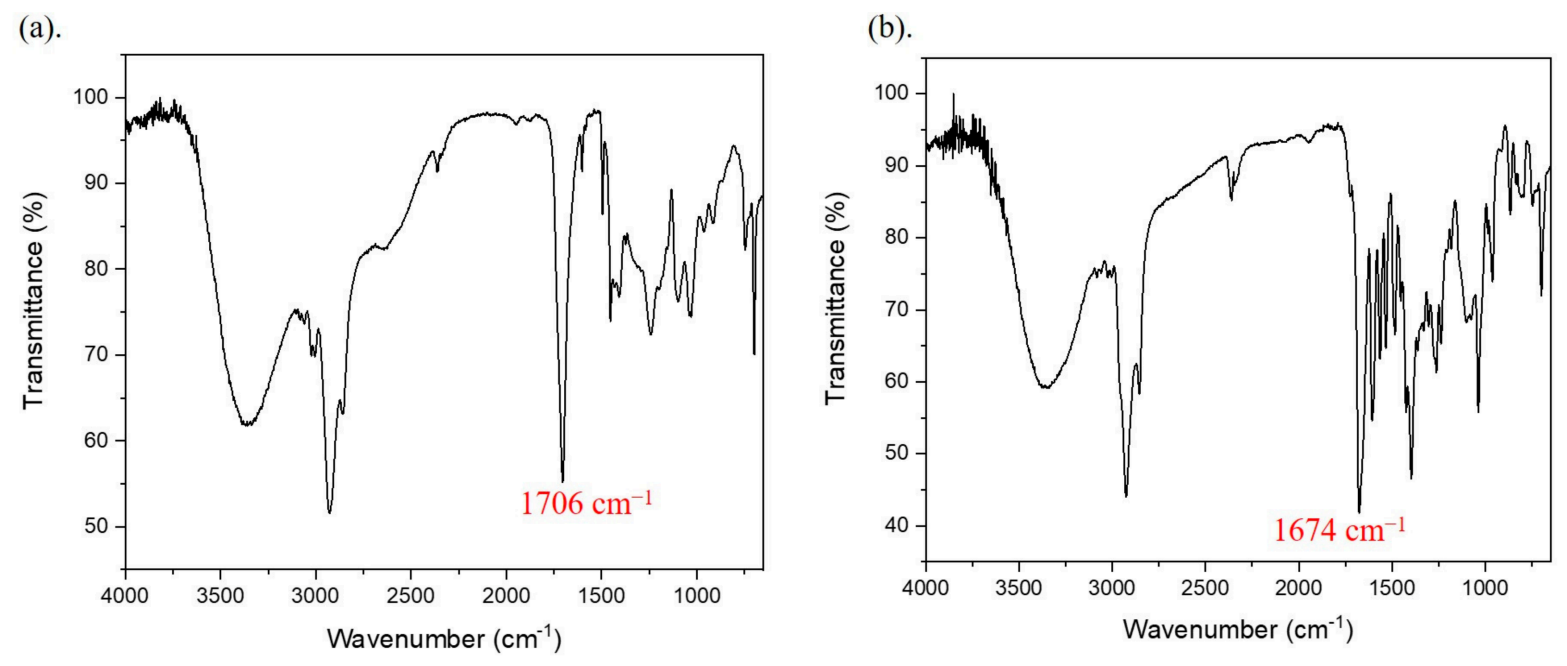
IR spectrum of (**a**) LPA and (**b**) LBJ-1.

The formation of amide bond was investigated through IR spectrum. Figure 2 shows that carboxylic acid C=O stretching peak at 1706 cm^−1^ shifted to amide characteristic peak at 1674 cm^−1^, indicating the success of amide formation in LBJ-1 synthesis.

**Figure 3:**
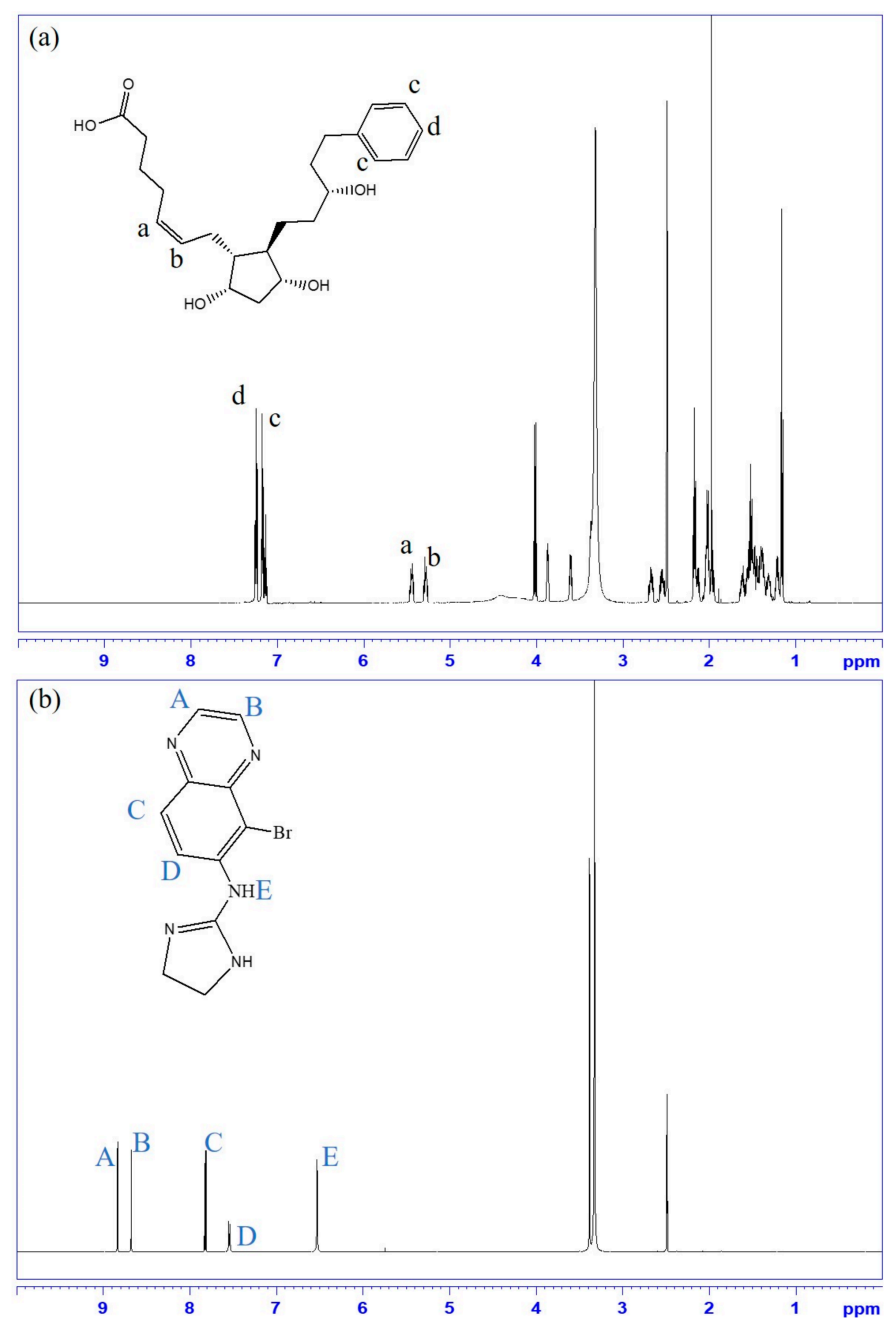
^1^H-NMR spectrum of (**a**) LPA and (**b**) BM. The lowercase letters and uppercase letters denote the corresponding protons in Table 1.Regioisomers of LBJ-1 (Figure 4) caused by two amine reaction sites are suspected in this synthesis; one is an amide bond forming at the imidazoline ring (iso-1-LBJ), and the other is an amide bond forming at the side chain (iso-2-LBJ). This hypothesis could be verified by the ^1^H-NMR spectrum of LBJ-1 (Figure 4). As compared to standard BM spectrum (b), four small peaks were observed from δ = 7.5 to 12 ppm green arrows, which might be attributed to the protons of quinoxaline from iso-2-LBJ (cite with *). These slight downfield shifts could be assigned to the carbonyl of amide formation near the quinoxaline group, which changes the electron densities of these protons. According to the integral areas for peaks of A and A*, the ratio of iso-1 and ios-2 is 10:1.

The identification of the LBJ-1 structure was based on the comparison of the ^1^H-NMR spectra of LPA (Figure 3a) and BM (Figure 3b) with that of LBJ-1. The characteristic symbols and corresponding chemical shifts for selected protons in LPA (a–d) and BM (A–E) are shown in Table 1.

**Figure 5:**
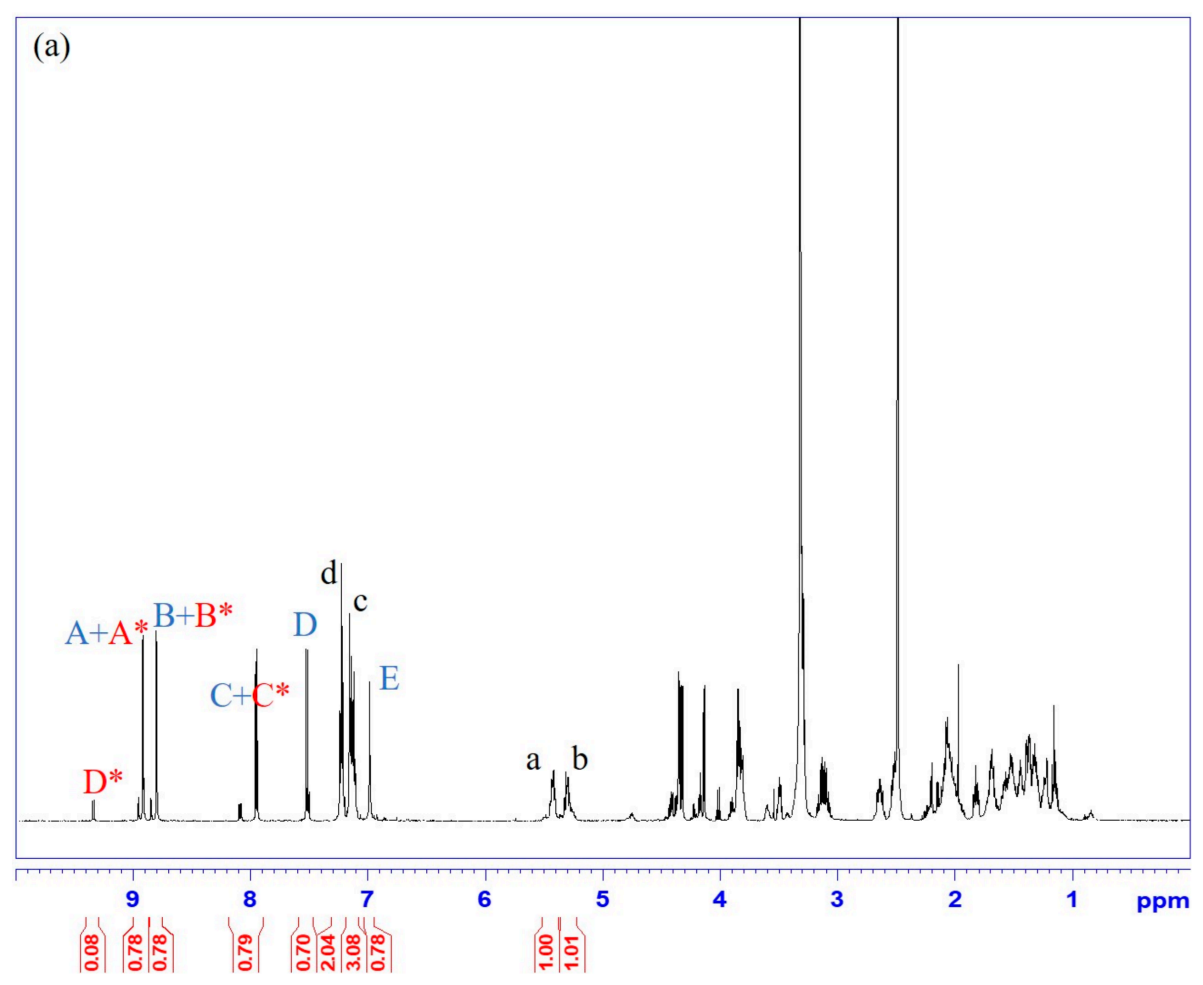

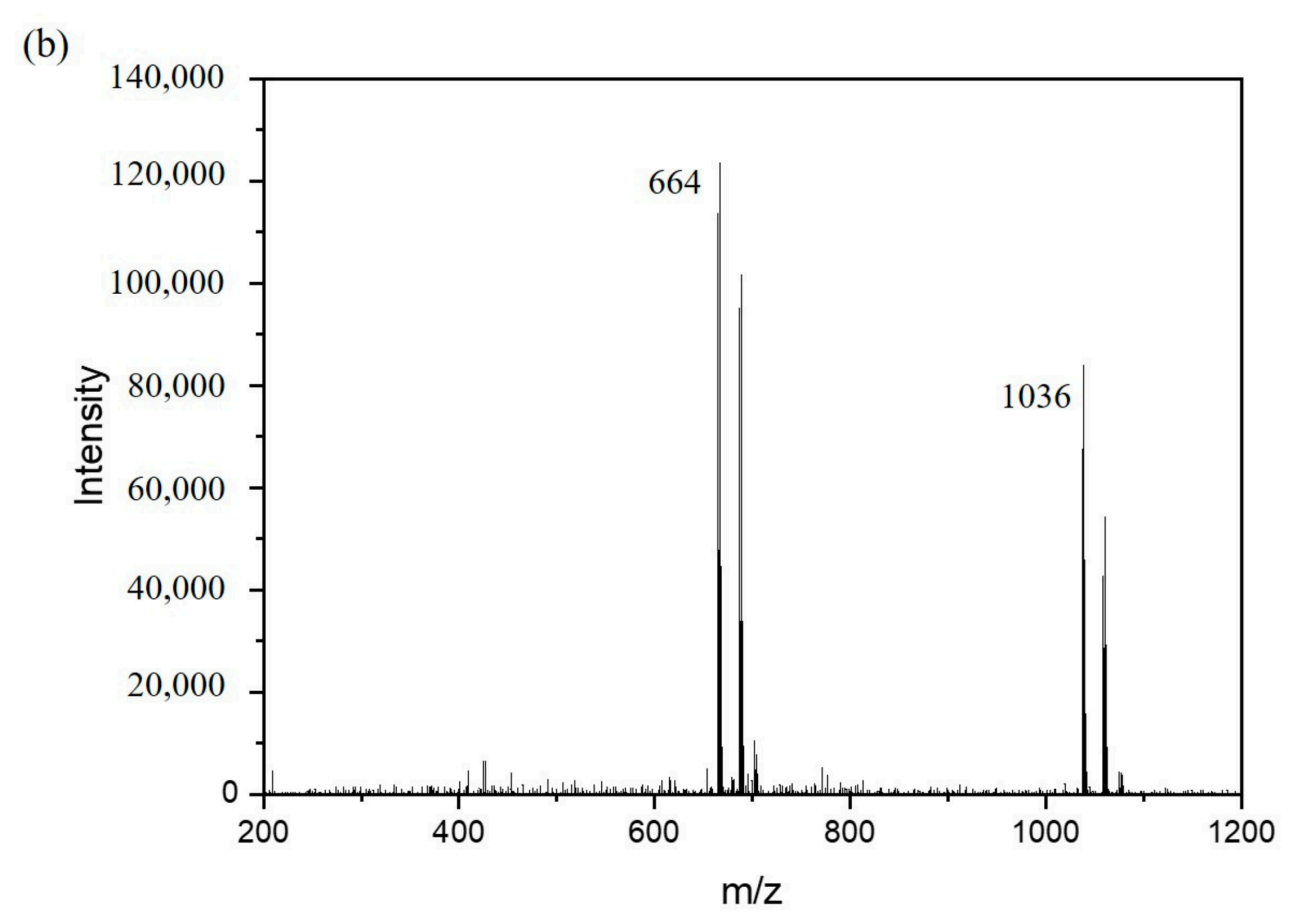
(**a**) ^1^H-NMR spectrum of LBJ-1, the lowercase letters and uppercase letters denote protons from LPA (Figure 3a) and BM (Figure 3b), respectively. (**b**) mass spectrum of LBJ-1.

On the other hand, ideally, one molecule of LPA reacted with one molecule of BM to form LBJ. However, the integral peak value shows that the ratio of LPA and BM was 1.25:1 (based on a: A + A* in Figure 5a), indicating more than one LPA participates in this reaction. One side product, LPA-LBJ (Figure S1), was suspected to be formed by the further coupling of the unprotected hydroxyl group of LBJ with the intermediate of Steglich esterification (LPA + EDC). This hypothesis is confirmed by the mass spectrum of LBJ-1 (Figure 5b), which shows two peaks corresponding to the molecular weights of LBJ (m/z = 664) and LPA-LBJ (m/z = 1036), respectively.

#### 2.1.2. Synthesis and Characterization of LBJ Through Method 2 (LBJ-2)

An alternative synthetic route for LBJ was proposed to avoid the undesired production of a side product through the same Steglich mechanism with the involvement of protecting agent (TBDMSCl) [28]. Herein, the hydroxyl groups in LP were initially protected with TBDMSCl, and imidazole acted as an organic base to capture the hydroxyl chloride produced during this process. The following hydrolysis process and amide formation were generally similar to method 1. Finally, the deprotection process was conducted with TBAF and quenched with CaCO_3_ and an ion exchanger [29].

**Figure 6:**
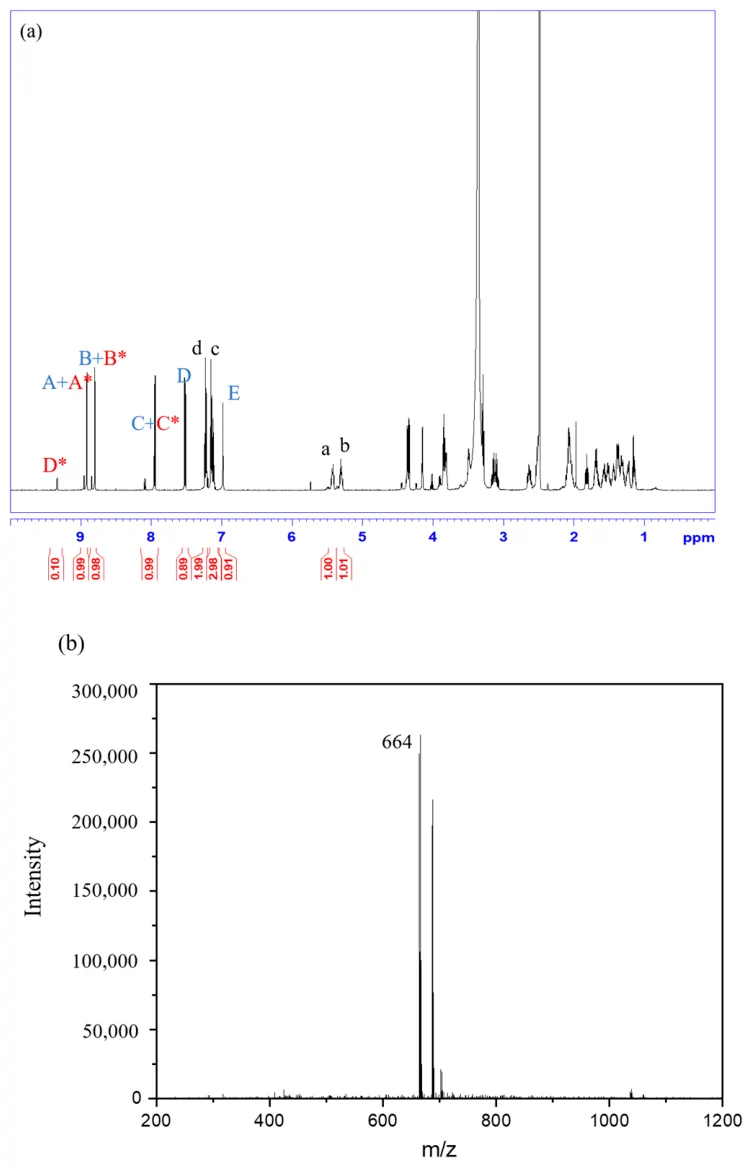
(**a**) NMR spectrum of LBJ-2, the lowercase and uppercase letters denote protons from LPA (Figure 3a) and BM (Figure 3b), respectively. (**b**) Mass spectrum of LBJ-2.

The protection process was analyzed by NMR spectra and is shown in Figures S2–S4. Briefly, the integral ratio of LPA and TBDMSCl was 1:3, which is consistent with three hydroxyl groups in LPA. In addition, NMR and mass results showed that the involvement of the protecting agent successfully prevents the formation of LPA-LBJ. Based on the integral values in the NMR spectrum of LBJ-2 (Figure 6a), the composition of LBJ-2 was determined to be LPA:BM = 1:1, indicating that pure LBJ without the ester side product was successfully synthesized. The identification of molecular weight of LBJ-2 was also performed by mass spectroscopy with electrospray ionization, and the result is shown in Figure 6b. Compared with the result of method 1, mass detection of LBJ-2 only showed the signal at m/z = 664, and without the signal corresponding to the undesired side product.

### 2.2. The HPLC Measurement Results and Calibration Curve

The calibration curves for quantitative analysis of LP, LPA, BM and, LBJ were established based on HPLC measurements, which were newly developed by us. The sample preparation method was based on the hydrolysis test shown in Section 3.5.

**Figure 7:**
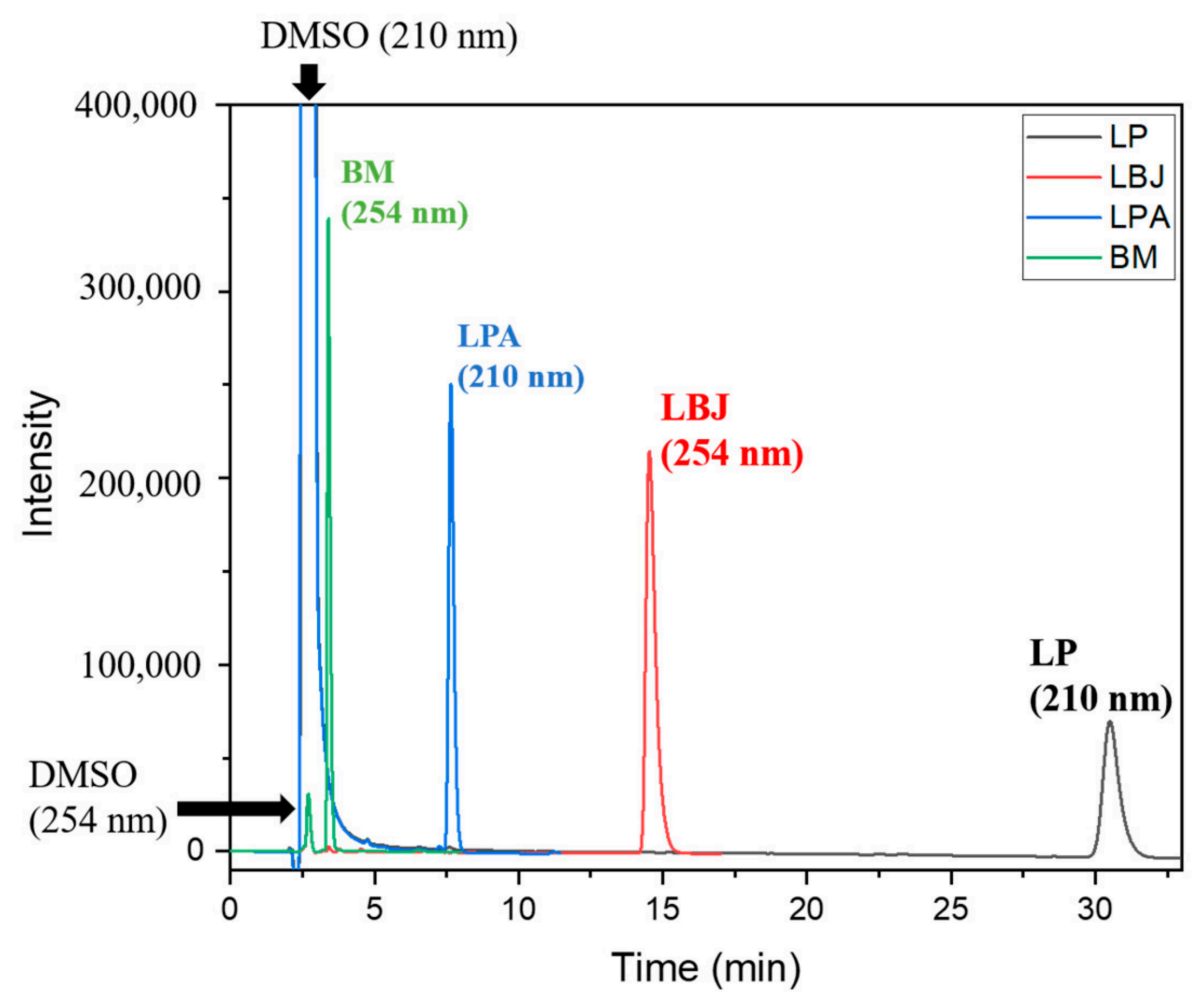
The HPLC spectrum of LP, LPA, BM and LBJ.

Figure 7 shows the HPLC spectrum of each sample and its corresponding retention time. The detection wavelength for BM and LBJ is 254 nm, while LPA and LP is 210 nm. The retention time of LP, LPA, BM and LBJ is 31.0 min, 7.8 min, 3.4 min, and 14.8 min, respectively. Calibration curves were constructed by plotting peak area against the concentration of each compound (ppm). Subsequently, samples with known concentrations (15, 25, and 45 ppm) were prepared to validate the calibration curve. Detailed information on the calibration curve and validation results were showed in the supporting information (Figures S5–S8, and Tables S1–S4).

### 2.3. In Vitro Hydrolysis Study

In glaucoma management, poor adherence has been consistently reported and is considered a major obstacle [30,31,32,33,34]. Enhancing education and communication is a common method to improve adherence [35,36]. Other methods, such as using preservative-free eye drops to decrease severe adverse effects [37] or switching to generic drugs, are potential alternatives for patients with economic problems [38].

However, the potential for patients to forget their medication is an uncontrollable, unpredictable factor that compromises efforts to improve adherence. To address this issue, sustained drug release systems or technologies are considered a feasible solution [39,40,41]. By prolonging the drug’s duration of action, reliance on patients’ adherence may be reduced. Here, the hydrolysis rate and duration of LBJ were studied to investigate the characteristics of a long-lasting API release profile.

#### 2.3.1. In Vitro Hydrolysis Test of LP

The in vitro test conditions for hydrolyzing LBJ are based on the pharmacokinetics (PK) results of LP in humans and hydrolase activity in ocular tissues. According to the prior research, after administration of LP eye drops, the concentration of LPA in aqueous humor reached its peak at 2.5 h and remained elevated for 4 h [42]. The transformation of LP to LPA could result from enzymatic activity. Carboxylesterases are associated with the hydrolysis of ester and amide compounds [43]. It was commonly observed in ocular tissues of humans and other species used for preclinical studies [44,45,46]. Therefore, the porcine liver esterase, which express multiple forms of carboxylesterases, is selected as a preliminary model for evaluating the in vitro hydrolysis studies [47].

**Figure 8:**
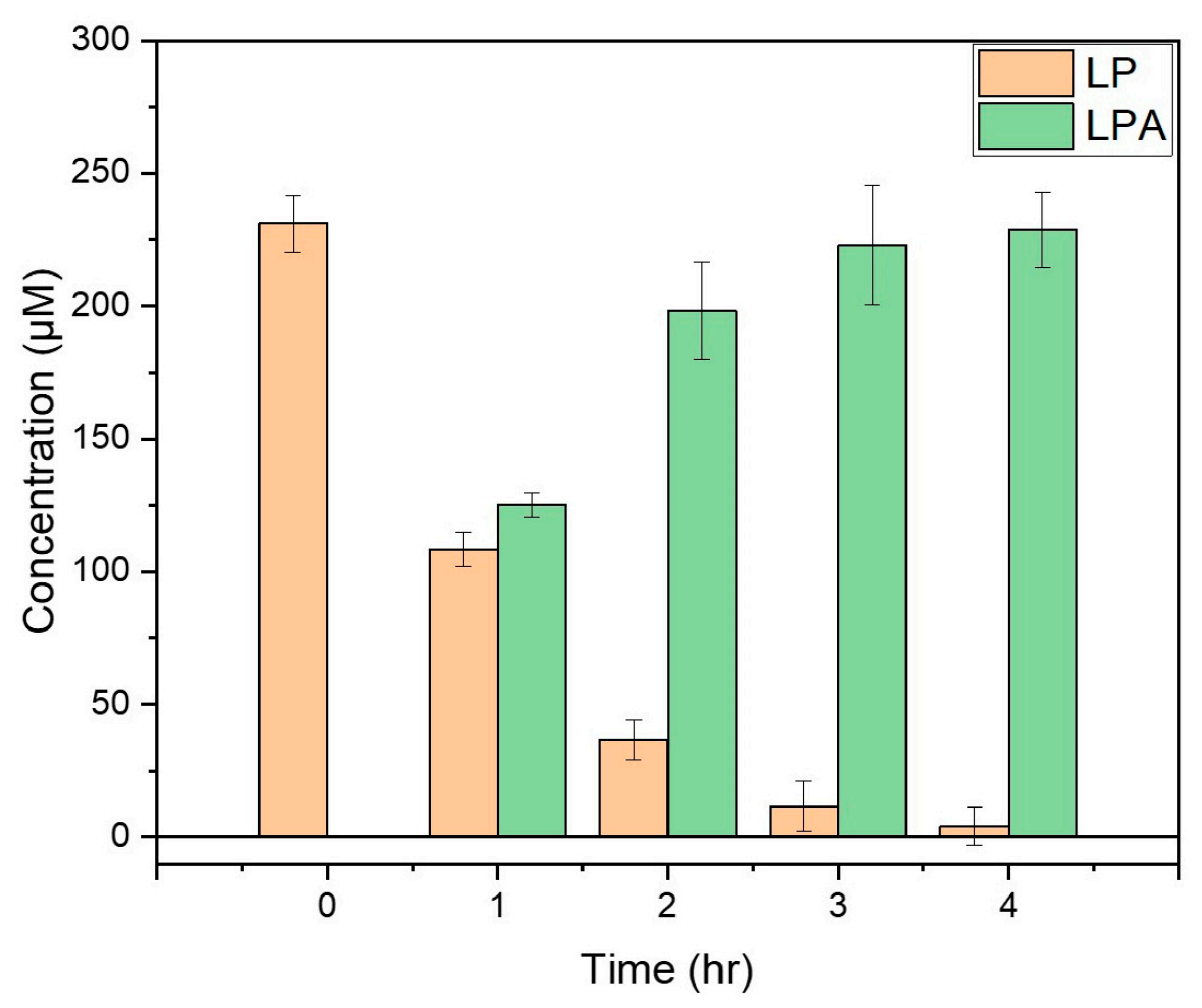
In vitro test of LP (100 ppm, in 10% DSMO) with 0.4 U/mL esterase in a 37 °C water bath.

Following the discovery of prior art, the esterase concentration was identified and validated using LP hydrolysis time in our lab. The result is shown in Figure 8. LP was almost completely hydrolyzed to LPA within 4 h at an esterase concentration of 0.4 U/mL. The complete datasets, including all individual replicate values, calculated means, HPLC, spectrum and standard deviations, have been provided in the Supporting Information (Table S5 and Figure S9).

#### 2.3.2. In Vitro Hydrolysis Test of LBJ

**Figure 9:**
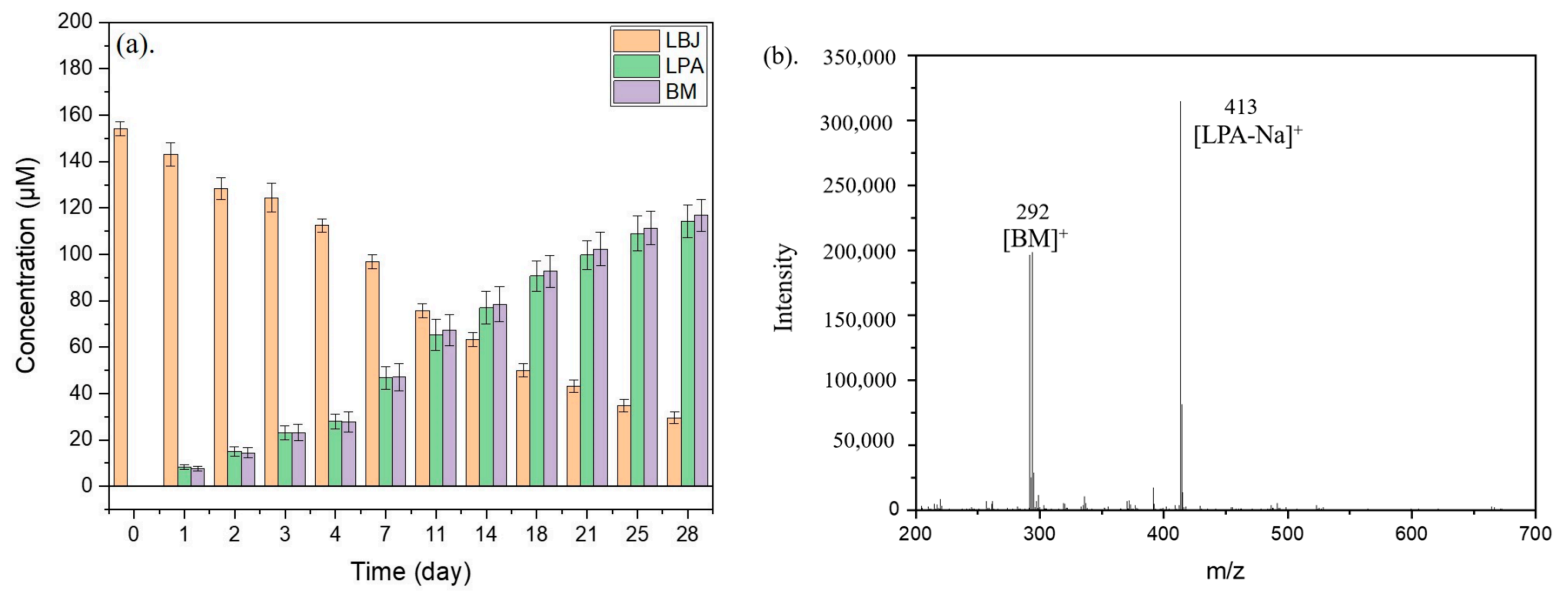
(**a**) In vitro test of LBJ (100 ppm, in 10% DSMO) with 0.4 U/mL esterase in 37 °C water bath and (**b**) mass spectrum of hydrolyzed products of LBJ.

The conditions for hydrolyzing LP were then applied to investigate the LBJ hydrolysis rate. The release of API, i.e., LPA and BM, started on day 1 and lasted for 28 days (Figure 9a). Compared to the LP’s 4 h duration, LBJ showed a prolonged hydrolysis time of 28 days. The concentration changes after 28 days of incubation are −124.67, 114.20, and 116.85 µM for LBJ, LPA, and BM, respectively. Mass spectrum was also used to analyze the hydrolysis products, as shown in Figure 9b. The signals at m/z = 292 were assigned to [BM]^+^ and at m/z = 413 were assigned to [LPA-Na]^+^, indicating successful transformation of the prodrug (LBJ) into APIs (LPA and BM). The complete datasets, including all individual replicate values, calculated means, HPLC spectrum and standard deviations, have been provided in the Supporting Information (Table S6 and Figure S10).

Sustained drug release systems are popular solutions for addressing the potential for patients to forget to administer their medication. Currently existing sustained drug release systems commonly rely on invasive implants, such as DurystaTM and iDose™. Durysta™ is an intracameral implant that steadily releases the prodrug bimatoprost to the ciliary body through the degradation of biodegradable polymers [48,49]. iDose^TM^ is an intracameral titanium implant that regulates the release of prodrug travoprost through biocompatible polymer membranes with different thicknesses [50].

In this work, a new hypothesis for achieving sustained release without an invasive implant is proposed and tested. By extending the hydrolysis time of a prodrug, the release of the API can be prolonged, leading to a potential application in longer IOP-lowering effect.

## 3. Materials and Methods

### 3.1. Materials

Latanoprost (LP, CAS number 130209-82-4 & batch number E028 235BB03), and latanoprost acid (LPA, CAS number 41639-83-2 & batch number C28M 255AA01) were purchased from Everlight Chemical, Taipei, Taiwan. Sodium hydroxide (NaOH, CAS number 1310-73-2 & lot number KZ-3243M) was purchased from Showa, Japan. Amberchrom^TM^ 50WX8 (hydrogen form, CAS number 69011-20-7 & catalog number 217514-100G), N-(3-Dimethylaminopropyl)-N′-ethylcarbodiimide hydrochloride (EDC, CAS number 25952-53-8 & catalog number E7750-5G), and esterase (from porcine liver, CAS number 9016-18-6 & catalog number E3019-3.5KU) were purchased from Sigma-Aldrich, St. Louis, MO, USA. Brimonidine (BM, CAS number 59803-98-4 & lot number A0470629), and Tetrabutylammonium fluoride (TBAF, 1M in THF, CAS number 429-41-4 & lot number A0449234) were purchased from Thermo Fisher Scientific, Loughborough, UK. 4-(Dimerthylamino) pyridine (DMAP, CAS number 1122-58-3 & lot number 10238027) was purchased from Thermo Fisher Scientific, Heysham, UK. Tert-butyldimethylchlorosilane (TBDMSCl, CAS number 18162-48-6 & lot number 10219364), and imidazole (CAS number 288-32-4 & lot number 10202197) were purchased from Alfa Aesar (Thermo Fisher Scientific), Heysham, UK. Magnesium sulfate (MgSO_4_) was purchased from REAGENTs DUKSAN, Ansan, Republic of Korea. Calcium carbonate (CaCO_3_) was purchased from J.T.Baker, Phillipsburg, NJ, USA.

### 3.2. Synthesis of LBJ ((5Z)-7-[(1R,2R,3R,5S)-3,5-Dihydroxy-2-[(1E,3S)-3-hydroxy-5-phenylpent-1-en-1-yl]cyclopentyl]-N-[5-bromo-N-(4,5-dihydro-imidazol-2-yl)quinoxalin-6-amine]-imidazole-1-yl-hept-5-enamide)

#### 3.2.1. Method 1 (LBJ-1)

LP (144.65 mg, 0.33 mmol) was dissolved in tetrahydrofuran (THF, 6.80 mL), followed by the addition of NaOH aqueous solution (1N, 3.40 mL) and ethanol (6.80 mL). The reaction mixture was stirred at room temperature for 1 day. After the hydrolysis reaction, the reaction mixture was neutralized with Amberchrom^TM^ 50WX8 Ion exchange resin (5220.00 mg). After neutralization, the solid residues were removed by filtration through Teflon filter (0.45 µm), and the filtrate was dried under reduced pressure. Then the crude product was dissolved in ethyl acetate (EA), and extracted with brine. After extraction, the organic layer was dried over MgSO_4_ to remove water. The MgSO_4_ was removed by filtration through a Teflon filter (0.45 µm). The filtrate was evaporated under reduced pressure and dried under vacuum. Gel-like LPA was obtained (132.23 mg, yield = 100.00%).

LPA (132.23 mg, 0.34 mmol, 1.0 eq) was dissolved in dichloromethane (DCM, 6.0 mL). Then, BM (138.27 mg, 0.47 mmol, 1.38 eq) and DMAP (124.14 mg, 1.01 mmol, 3 eq) were added to the LPA solution. After stirring at room temperature for 10 min, EDC (129.91 mg, 0.68 mmol, 2 eq, in 2.0 mL DCM) was added to the reaction mixture. After overnight reaction at room temperature, the reaction mixture was extracted with water. The organic layer was collected and dried over MgSO_4_. Then, MgSO_4_ was removed by filtration through a Teflon filter (0.45 μm), and filtrate was concentrated under reduced pressure c. The crude product was further purified by column chromatography (stationary phase: silica gel, mobile phase: EA/methanol = 5:1) and obtained LBJ-1 (77.08 mg, yield = 34.24%). The synthesis was independently repeated three times. The isolated yields of the other two runs were 30.75% and 38.68%, respectively.

#### 3.2.2. Method 2 (LBJ-2)

LP (219.82 mg, 0.51 mmol, 1eq) was dissolved in DCM (9.0 mL), and then added imidazole (692.84 mg, 10.18 mmol, 20 eq) and TBDMSCl (766.98 mg, 5.10 mmol, 10 eq). The reaction mixture was stirred at room temperature overnight. After reaction, the solid precipitate was removed by filtration (Teflon, 0.45 μm). The filtrate was concentrated under reduced pressure and purified by column chromatography (stationary phase: silica gel, mobile phase: EA/hexane = 10:1). The gel-like product, Latanoprost-TBMDMSCl (LP-T, 365.00 mg, yield = 100.00%), was obtained.

LP-T (362.05 mg, 0.221 mmol) was dissolved in THF (5.70 mL), and then NaOH aqueous solution (1N, 2.85 mL) and ethanol (5.70 mL) were added. The reaction mixture was stirred at room temperature for 1 day. After hydrolysis, the reaction mixture was neutralized by Amberchrom^TM^ 50WX8 Ion exchange resin (4543.00 mg). After neutralization, the solid residue was removed by filtration (Teflon, 0.45 μm), and the filtrate was concentrated under reduced pressure. The resulting gel-like crude product was dissolved in DCM, and extracted with water. The organic layer was dried over MgSO_4_, filtered (Teflon, 0.45 μm), and concentrated under reduced pressure. The product, LPA-TBDMSCl (LPA-T, 303.92 mg, yield = 88.68%) was obtained.

LPA-T (163.79 mg, 0.22 mmol, 1.0 eq) was dissolved in DCM (5.5 mL). Then BM (78.34 mg, 0.27 mmol, 1.23 eq) and DMAP (81.38 mg, 0.67 mmol, 3 eq) were added to LPA-T solution. After stirring at room temperature for 10 min, EDC (85.17 mg, 0.44 mmol, 2 eq, in 2.0 mL DCM) was added to the reaction mixture. After stirring overnight at room temperature, the reaction mixture was extracted with water. The organic layer was collected and dried over MgSO_4_. The drying agent was removed by filtration (Teflon, 0.45 μm), and the filtrate was concentrated under reduced pressure. The crude product was further purified by column chromatography (stationary phase: silica gel, mobile phase: EA/hexane = 1:1) and obtained LBJ-TBDMSCl (LBJ-T, 127.13 mg, yield = 56.50%).

LBJ-T (125.28 mg, 0.12 mmol, 1 eq) was dissolved in THF (3.0 mL) and placed in a 10 °C water bath. TBAF (1M, 1.12 mL, 1.12 mmol, 9 eq) was added to LBJ-T solution. The deprotection process was carried out at room temperature for 4 days. CaCO_3_ (1736.32 mg) and Amberchrom^TM^ 50WX8 Ion exchange resin (1736.32 mg) were used to quench the deprotection process. The reaction mixture was filtered, concentrated under reduced pressure, and extracted with DCM/water. The crude product was further purified by column chromatography (stationary phase: silica gel, mobile phase: EA/methanol = 5:1) to afford LBJ-2 (32.52 mg, yield = 39.35%). The synthesis was independently repeated three times. The isolated yields of the other two runs were 30.75% and 26.43%, respectively.

### 3.3. Analysis

#### 3.3.1. Nuclear Magnetic Resonance (NMR)

The NMR measurements were performed by the instrument center of National Cheng Kung University. Model: Bruker AVANCE III HD 600MHz NMR Spectrometer. Solvent: Dimethyl sulfoxide-d (DMSO-d).

#### 3.3.2. Attenuated Total Reflectance (ATR)

The ATR was conducted with infrared spectroscopy. Model: Nicolet 6700, Thermo Scientific (Cambridge, UK).

#### 3.3.3. High-Performance Liquid Chromatograph (HPLC) and Mass Spectrometer (MS)

Both spectra were measured using a SHIMADZU LC-2050C equipped with different columns. For HPLC, ODS-100V, 4.6 × 250 mm, purchased from TOSOH; mobile phase: water (0.025% formic acid)/Acetonitrile = 55:45, used in the calibration curve and hydrolysis test; flow rate: 1 mL/min, detector: UV-vis photodiode array detector. For mass detection, Super-ODS, 2.0×100 mm, purchased from TOSOH, mobile phase: water (0.025%, formic acid)/Acetonitrile = 40:60, flow rate: 0.2 mL/min, detector: electrospray ionization (ESI), ionization mode: positive, scan range: m/z 200–800 with 10 ppm sample concentration in ACN, 800–1200 with 100 ppm sample concentration in ACN.

### 3.4. Calibration Curve

The calibration curve of LP, LPA, BM and LBJ were obtained from HPLC measurement. The peak area was plotted as y axis, and sample concentration (ppm) was plotted as x axis.

All samples were initially prepared at 120 ppm in 10% DMSO and then diluted to 100, 80, 60, 40, 20 ppm with 10% DMSO. Before HPLC analysis, 1 mL of each sample was mixed with 1 mL of acetonitrile (ACN) and filtered through a Teflon filter (0.45 μm).

The detection wavelength for LP and LPA was 210 nm, whereas that for BM and LBJ was 254 nm.

### 3.5. Enzymatic Hydrolysis Test

An in vitro enzymatic hydrolysis was conducted using esterase from porcine liver (0.4 U/mL, 16 units/mg). The samples included LP (*n* = 3), and LBJ-2 (*n* = 3). The initial sample was 100 ppm in 10% DMSO. The incubation temperature was 37 °C. Before HPLC analysis, 0.5 mL of each sample was mixed with 0.5 mL of ACN and filtered through a Teflon filter (0.45 μm).

## 4. Conclusions

An amide prodrug that consists of BM and LPA was synthesized. A slower hydrolysis rate was achieved by modifying the ester bond (LP) to an amide bond (LBJ). LBJ could be synthesized through two methods, including Steglich esterification with or without the use of the protecting agent (TBDMSCl). In the method without the use of the protecting agent, LBJ may further react with LPA to form an ester by-product, as indicated by the NMR integral values. Instead of the theoretical ratio of 1:1, the integral ratio between LP and BM was 1.25:1, suggesting the formation of an undesired by-product (LPA-LBJ). To address this problem, TBDMSCl was introduced to protect the hydroxyl group from reacting with carbonyl acid intermediate, and this process successfully afforded LBJ without an unexpected by-product.

Under the enzymatic conditions, the in vitro hydrolysis rate of LBJ was much slower in comparison to LP. It took 28 days for LBJ to be completely hydrolyzed, while it took only 4 h to do the same to LP. LPA and BM, two anti-glaucoma APIs, were consistently formed and released to the environment during the 28-day study period.

LBJ is a promising candidate for a sustained API release system. When LBJ is formulated as an eye drop, a single dose every several days may become feasible. The interruption of the daily dosing due to potential for patients to forget their medication is more forgiving. Moreover, when LBJ is formulated with appropriate drug delivery carriers, enhancing corneal retention is expected for LBJ, which potentially leads to a longer IOP-lowering effect [51,52]. However, further studies, including ocular safety evaluation, aqueous humor drug concentration profiling, and IOP-lowering efficacy assessment, are required to determine whether these advantages can be translated into the ocular environment.

## Figures and Tables

**Figure 4:**
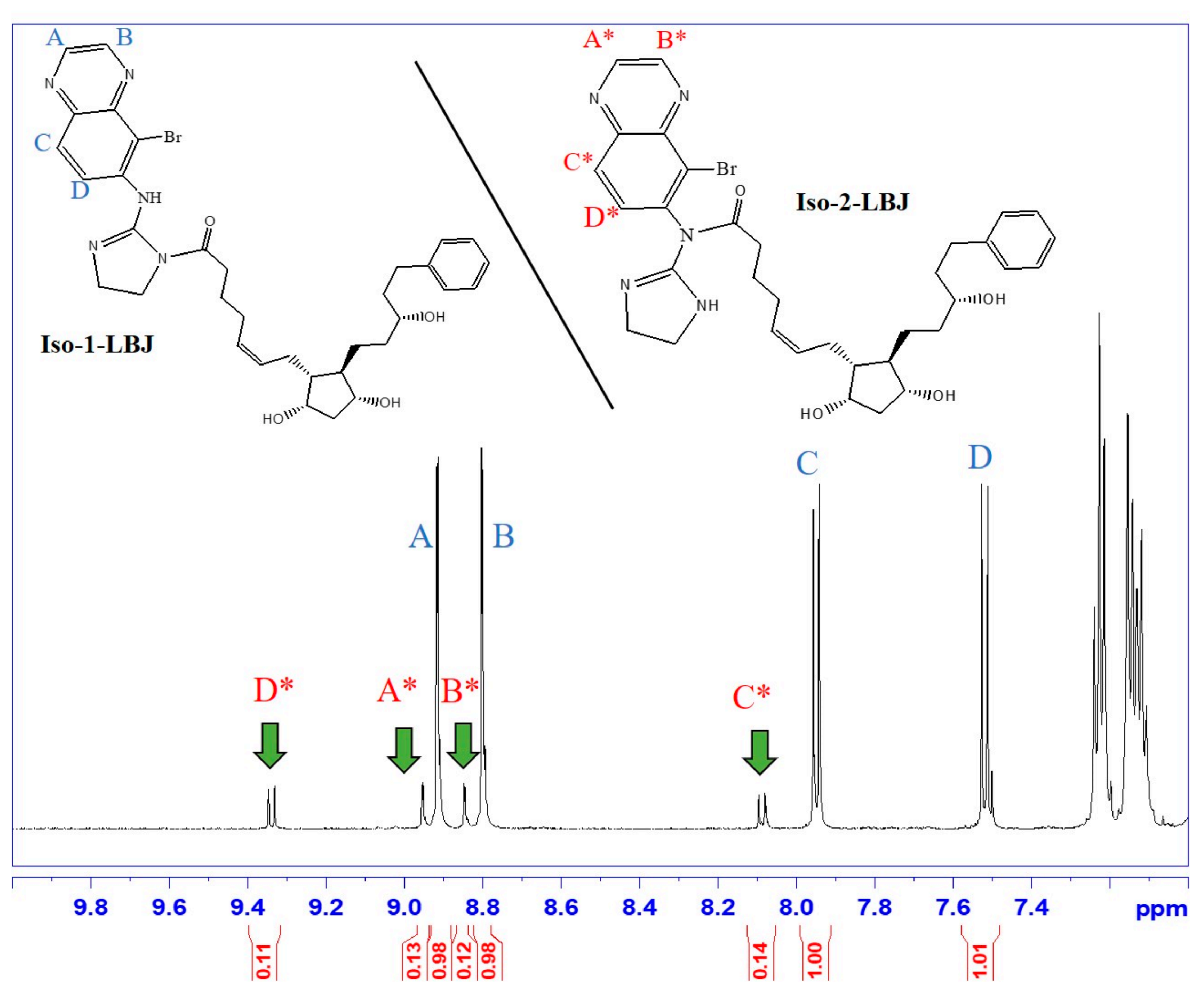
The regioisomers of LBJ. The uppercase letters with blue colors denote to iso-1-LBJ, and uppercase letters with red colors denote to iso-2-LBJ.

**Table 1:** The characteristics of ^1^H-NMR spectra of LPA and BM.

Compound	LPA	LPA	BM	BM
Symbol	a, b	c, d	A–D	E
Porton site	vinyl group	benzene	quinoxaline	amine
Chemical shift (δ, ppm)	5.3, 5.5	7.2, 7.3	8.9, 8.7, 7.8, 7.6	6.5

## Data Availability

The data presented in this study are available from the corresponding author upon reasonable request.

## Supplementary Materials

The following supporting information can be downloaded at: https://www.mdpi.com/article/10.3390/molecules31162780/s1, Figure S1. The structure of ester side product, LBJ-LPA; Figure S2. NMR spectrum of LP-T. Integral area of “a” from latanoprost and “p1”, “p2” from TBDMSCl are used to identify the chemical structure. Theoretically, the ratio of a:p1:p2 = 1:18:27, but in this result, excess integral area of protection agent means that unreacted TBDMSCl remained in LP-T. However, this problem can be resolved in second step. From the NMR result of LPA-T (Figure S3), the integral ratio becomes a:p1:p2 = 1:18:27, Figure S3. NMR spectrum of LPA-T; Figure S4. NMR spectrum of LBJ-T. The integral ratio between LPA part (a) and BM part (A+A*) is 1:1, means that protection process can prevent the unexpected side product from forming; Figure S5. The calibration curve of LP; Figure S6. the calibration curve of LPA; Figure S7. The calibration curve of BM; Figure S8. the calibration curve of LBJ; Table S1. The validation test of LP calibration curve. Table S2. the validation test of LPA calibration curve; Table S3. The validation test of BM calibration curve; Table S4. The validation test of LBJ calibration curve; Table S5. The raw data, calculated means, and standard deviations of LP hydrolysis; Figure S9. HPLC spectrum of LP hydrolysis; Table S6. The raw data, calculated means, and standard deviations of LBJ hydrolysis; Figure S10. HPLC spectrum of LBJ hydrolysis with (a) 254 nm and (b) 210 nm detection wavelength.
