# Supplementary material for: Latanoprost Acid–Brimonidine, a New Amide Prodrug for Glaucoma Management Based on the Concept of Sustained Release
Source: Molecules. 2026 Aug 10;31(16):2780. doi: 10.3390/molecules31162780 (PMC13515571; doi:10.3390/molecules31162780)
Supplement: Supplementary file 1 [file molecules-31-02780-s001.zip › molecules-4430365-supplementary.pdf]

Supporting information

# Latanoprost Acid–Brimonidine, a New Amide Prodrug for Glaucoma Management Based on the Concept of Sustained Release

Hong-Jia Lin [1], Shih-Horng Su [2] and Wen-Chung Wu [1,*]

[1] Department of Chemical Engineering, National Cheng Kung University, Tainan 701, Taiwan

2 DuNing Incorporated, Tustin, CA 92780, USA

* Corresponding author: wcwu@mail.ncku.edu.tw

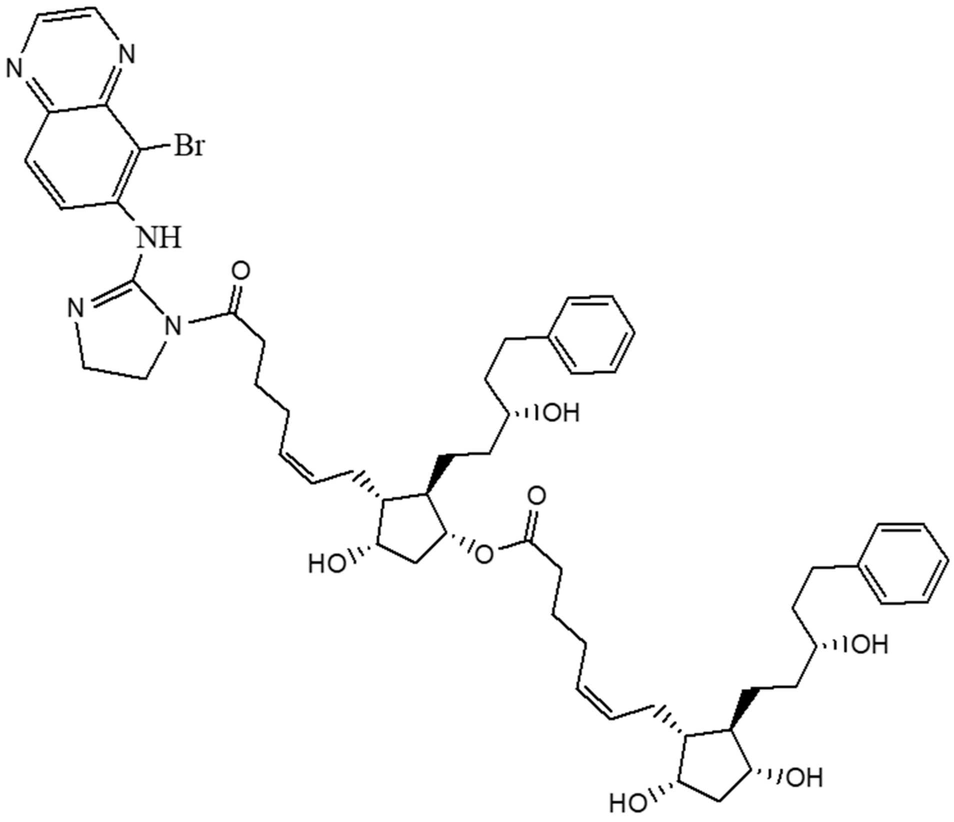


Figure S1. The structure of ester side product, LBJ-LPA

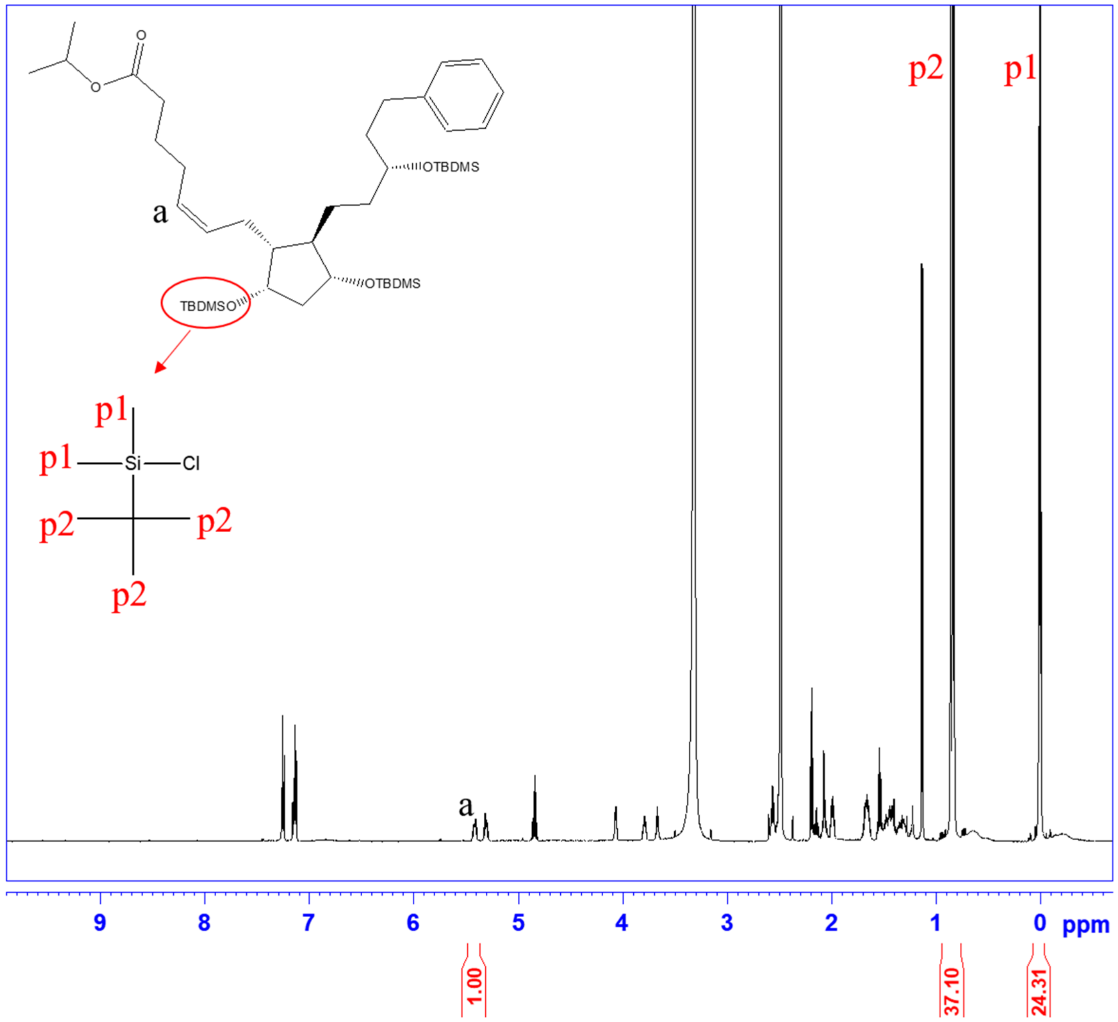


Figure S2. NMR spectrum of LP-T. Integral area of "a" from latanoprost and "p1", "p2" from TBDMSCl are used to identify the chemical structure. Theoretically, the ratio of a: p1: p2 = 1: 18: 27, but in this result, excess integral area of protection agent means that unreacted TBDMSCl remained in LP-T. However, this problem can be resolved in second step. From the NMR result of LPA-T (Figure S3), the integral ratio becomes a: p1: p2 = 1: 18: 27.

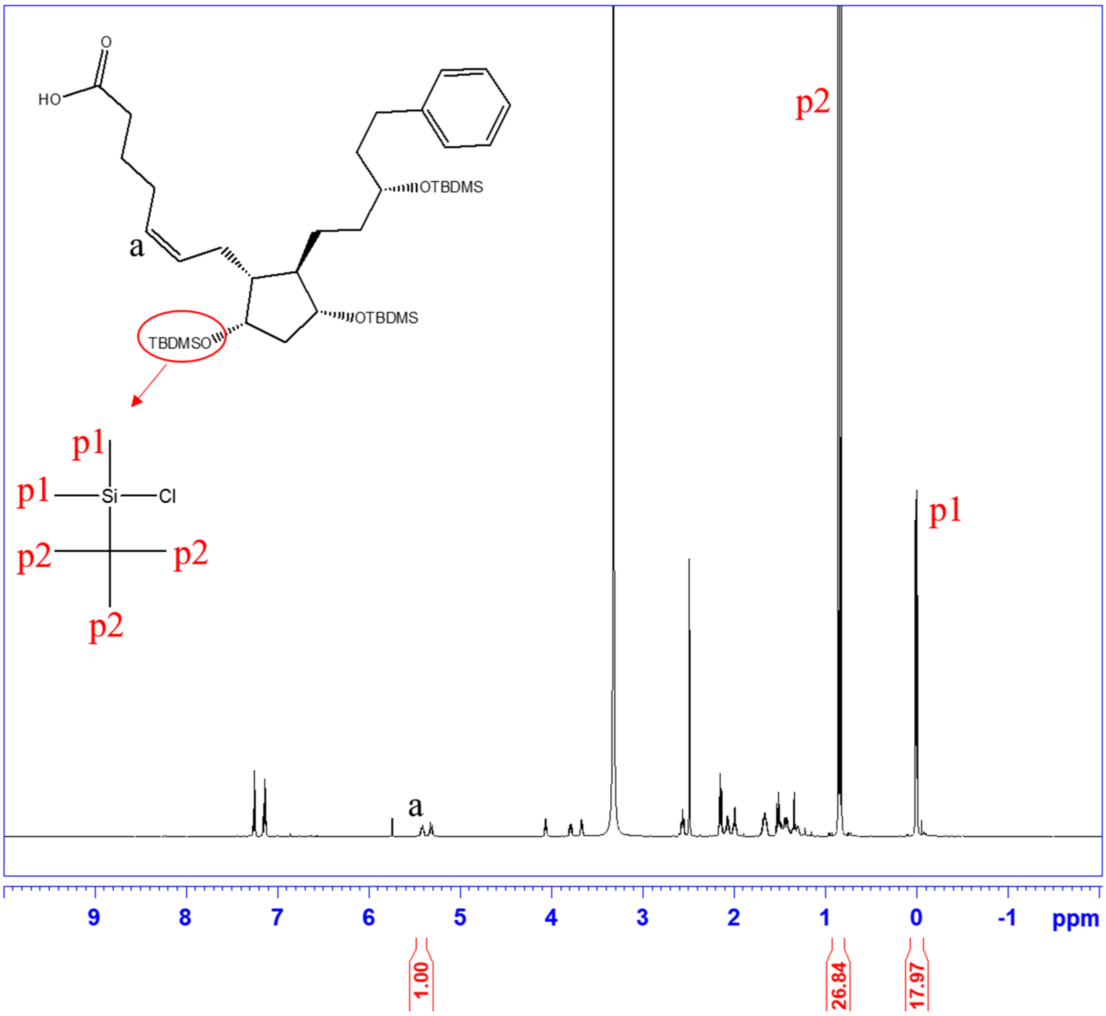


Figure S3. NMR spectrum of LPA-T

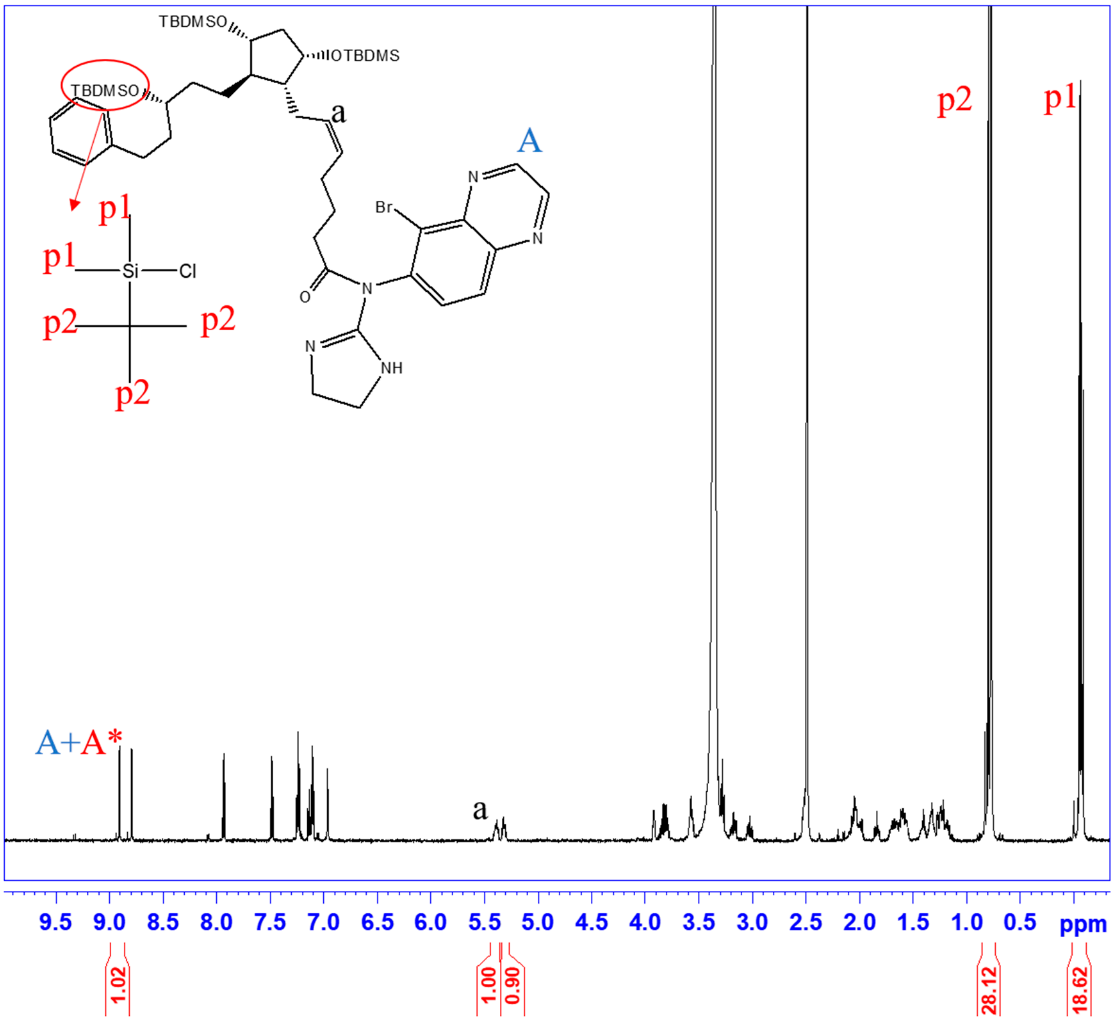


Figure S4. NMR spectrum of LBJ-T. The integral ratio between LPA part (a) and BM part (A+A*) is 1:1, means that protection process can prevent the unexpected side product from forming.

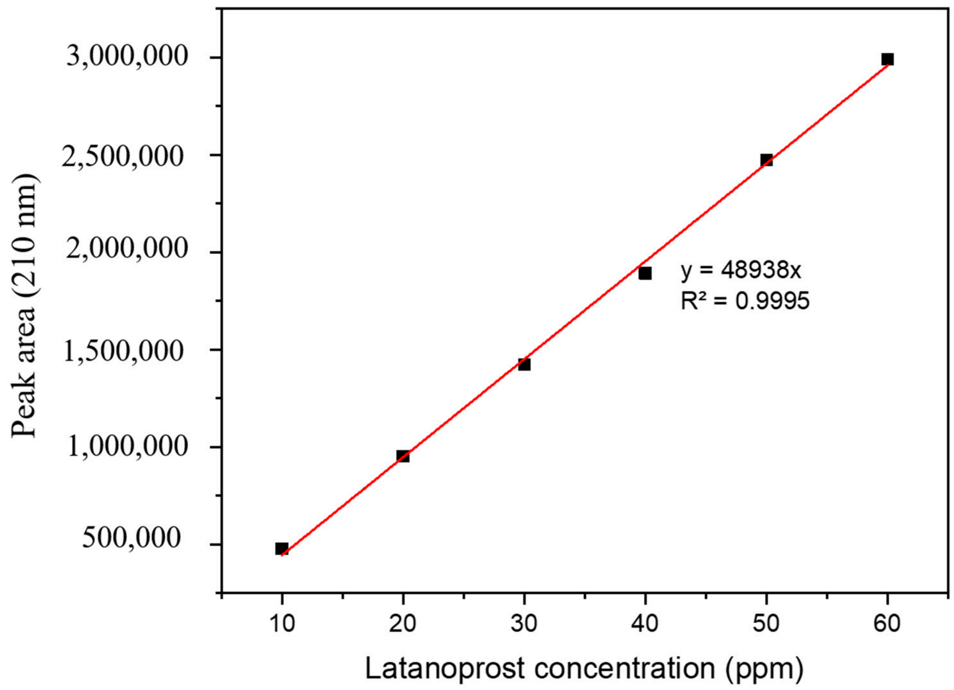


Figure S5. The calibration curve of LP.

Table S1. The validation test of LP calibration curve

| Compound | Known concentration (ppm) | Calculation concentration (ppm) |
|---|---|---|
| LP | 15.00 | 15.71 |
| | 25.00 | 25.69 |
| | 45.00 | 47.00 |

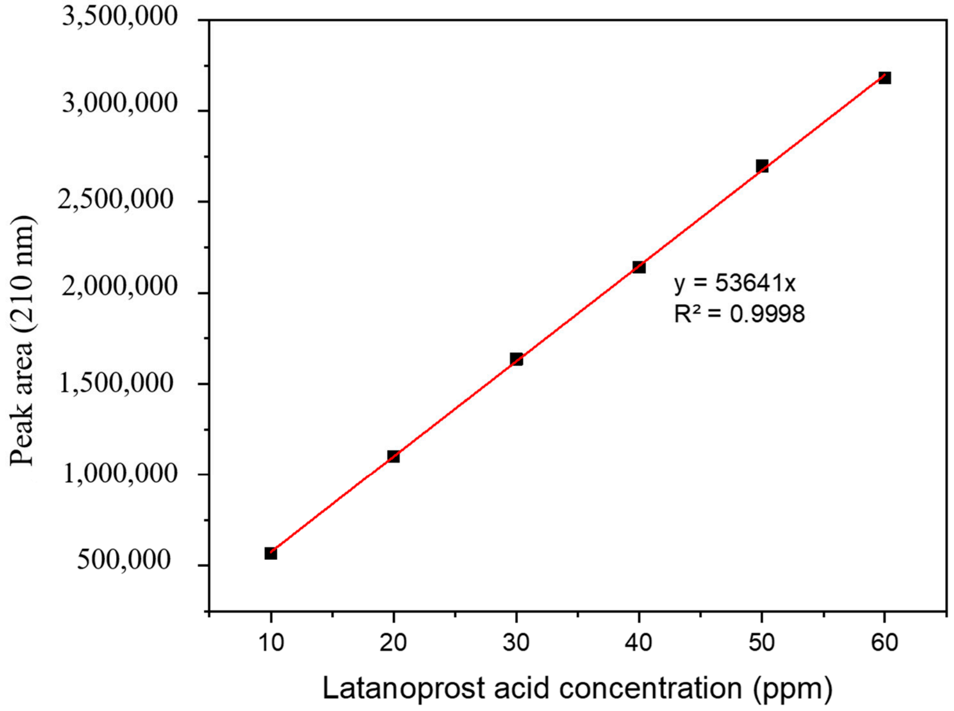


Figure S6. The calibration curve of LPA.

Table S2. The validation test of LPA calibration curve

| Compound | Known concentration (ppm) | Calculation concentration (ppm) |
|---|---|---|
| LPA | 15.00 | 15.18 |
| | 25.00 | 27.38 |
| | 45.00 | 45.75 |

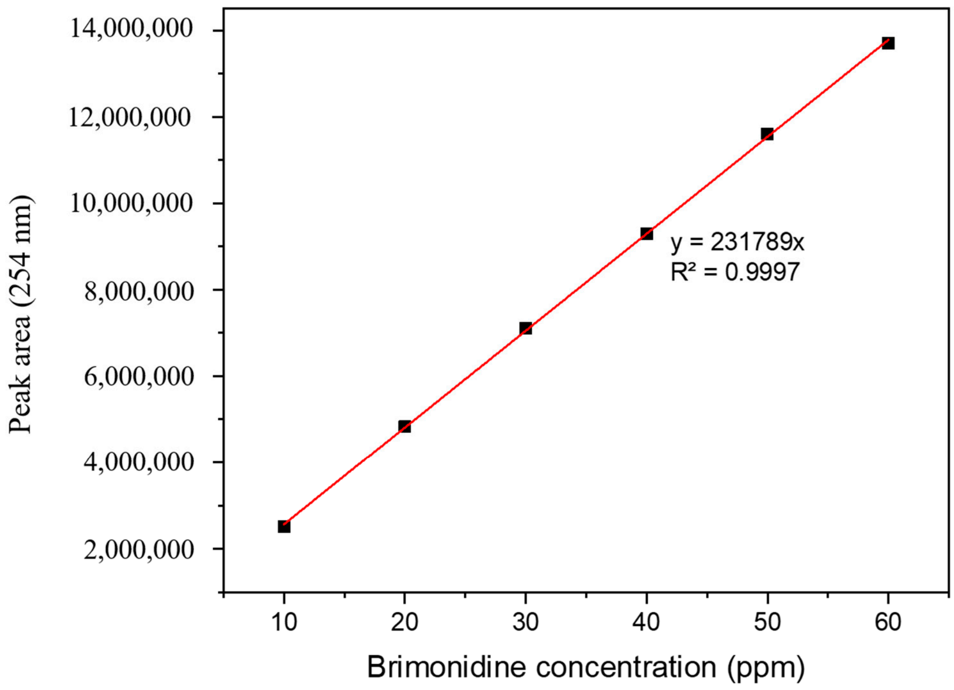


Figure S7. The calibration curve of BM.

Table S3. The validation test of BM calibration curve

| Compound | Known concentration (ppm) | Calculation concentration (ppm) |
|---|---|---|
| BM | 15.00 | 16.80 |
| | 25.00 | 27.14 |
| | 45.00 | 47.34 |

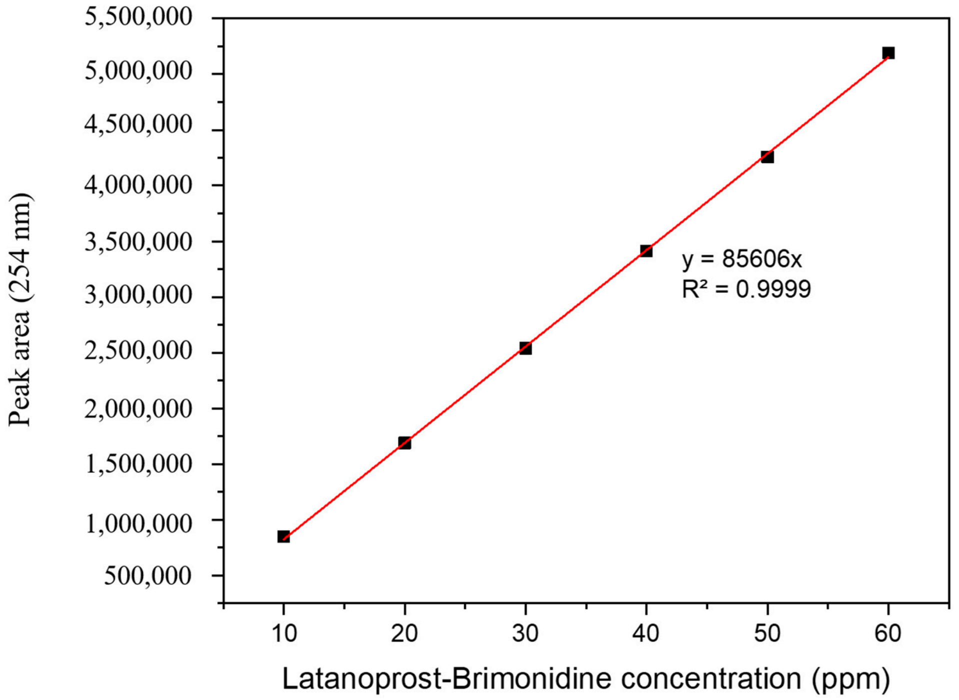


Figure S8. The calibration curve of LBJ.

Table S4. The validation test of LBJ calibration curve

| Compound | Known concentration (ppm) | Calculation concentration (ppm) |
|---|---|---|
| LBJ | 15.00 | 15.21 |
| | 25.00 | 25.34 |
| | 45.00 | 45.12 |

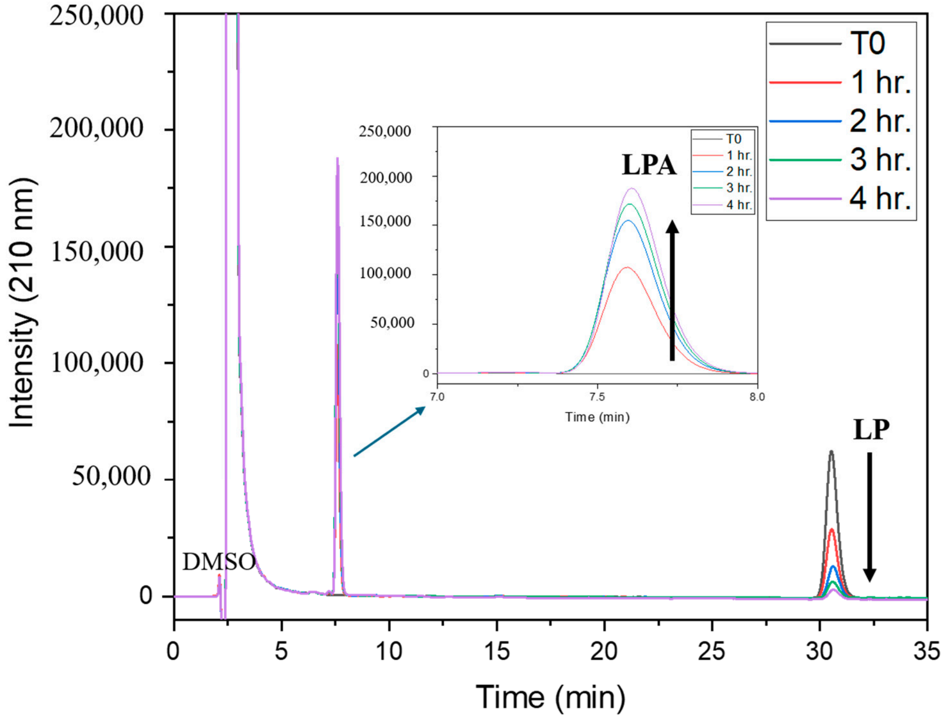


Figure S9. HPLC spectrum of LP hydrolysis

Table S5. The raw data, calculated means, and standard deviations of LP hydrolysis

| **LP (µM)** | 1st | 2nd | 3rd | AVG | STD |
|---|---|---|---|---|---|
| T0 | 218.82 | 235.13 | 238.99 | 230.98 | 10.71 |
| 1h | 101.18 | 112.57 | 111.37 | 108.37 | 6.26 |
| 2h | 45.61 | 33.12 | 31.39 | 36.71 | 7.76 |
| 3h | 22.60 | 6.80 | 5.42 | 11.61 | 9.55 |
| 4h | 12.59 | 0.00 | 0.00 | 4.20 | 7.27 |
| | | | | | |
| **LPA (µM)** | 1st | 2nd | 3rd | AVG | STD |
| T0 | 0.00 | 0.00 | 0.00 | 0.00 | 0.00 |
| 1h | 121.54 | 123.77 | 130.30 | 125.20 | 4.55 |
| 2h | 176.86 | 208.29 | 209.03 | 198.06 | 18.36 |
| 3h | 196.94 | 234.66 | 237.34 | 222.98 | 22.59 |
| 4h | 212.16 | 235.83 | 238.22 | 228.74 | 14.41 |

(a)

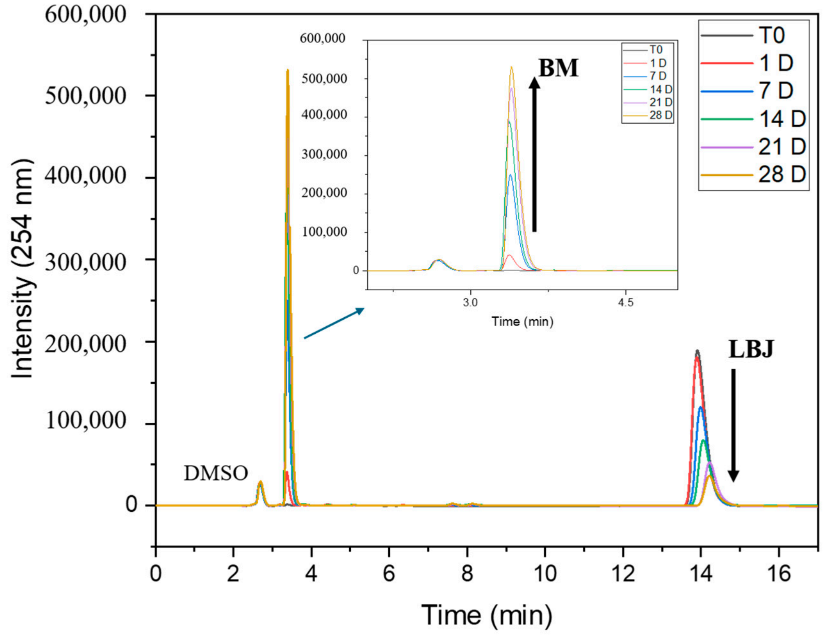


(b)

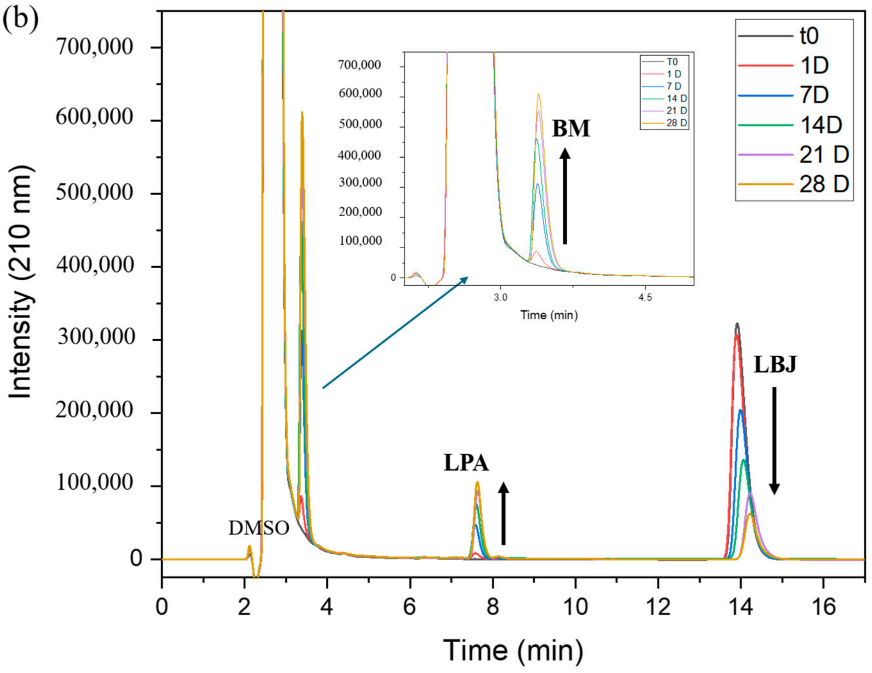


Figure S10. HPLC spectrum of LBJ hydrolysis with (a) 254 nm and (b) 210 nm detection wavelength

Table S6. The raw data, calculated means, and standard deviations of LBJ hydrolysis

| **LBJ (μM)** | 1st | 2nd | 3rd | AVG | STD |
|---|---|---|---|---|---|
| T0 | 151.93 | 153.14 | 157.47 | 154.18 | 2.91 |
| 1D | 141.73 | 139.08 | 148.75 | 143.19 | 5.00 |
| 2D | 129.8 | 123.20 | 132.01 | 128.34 | 4.58 |
| 3D | 127.39 | 117.20 | 128.84 | 124.48 | 6.34 |
| 4D | 114.00 | 109.29 | 114.22 | 112.50 | 2.79 |
| 7D | 95.62 | 100.07 | 94.69 | 96.79 | 2.88 |
| 11D | 75.71 | 78.89 | 72.92 | 75.84 | 2.99 |
| 14D | 61.19 | 66.82 | 62.14 | 63.38 | 3.01 |
| 18D | 47.99 | 53.23 | 48.76 | 49.99 | 2.83 |
| 21D | 41.55 | 46.24 | 41.91 | 43.23 | 2.61 |
| 25D | 33.16 | 38.07 | 33.45 | 34.89 | 2.75 |
| 28D | 27.83 | 32.35 | 28.34 | 29.51 | 2.48 |
| | | | | | |
| **LPA (μM)** | 1st | 2nd | 3rd | AVG | STD |
| T0 | 0.00 | 0.00 | 0.00 | 0.00 | 0.00 |
| 1D | 9.19 | 7.13 | 8.23 | 8.18 | 1.03 |
| 2D | 16.27 | 12.89 | 16.03 | 15.06 | 1.89 |
| 3D | 24.46 | 19.56 | 25.04 | 23.02 | 3.01 |
| 4D | 28.05 | 24.74 | 31.14 | 27.98 | 3.20 |
| 7D | 46.83 | 41.74 | 51.69 | 46.75 | 4.98 |
| 11D | 66.13 | 58.34 | 71.75 | 65.41 | 6.73 |
| 14D | 77.43 | 69.75 | 83.85 | 77.01 | 7.06 |
| 18D | 89.26 | 85 | 97.68 | 90.65 | 6.45 |
| 21D | 99.04 | 93.72 | 106.31 | 99.69 | 6.32 |
| 25D | 105.72 | 103.7 | 117.67 | 109.03 | 7.55 |
| 28D | 111.08 | 109.31 | 122.23 | 114.21 | 7.00 |
| | | | | | |
| **BM (μM)** | 1st | 2nd | 3rd | AVG | STD |
| T0 | 0.00 | 0.00 | 0.00 | 0.00 | 0.00 |
| 1D | 8.28 | 6.50 | 8.20 | 7.66 | 1.01 |
| 2D | 15.29 | 12.01 | 16.14 | 14.48 | 2.18 |
| 3D | 24.14 | 19.16 | 26.13 | 23.14 | 3.59 |
| 4D | 28.81 | 22.9 | 31.55 | 27.75 | 4.42 |
| 7D | 47.84 | 40.78 | 52.68 | 47.10 | 5.98 |

| | | | | | |
|---|---|---|---|---|---|
| 11D | 68.51 | 60.02 | 73.44 | 67.32 | 6.79 |
| 14D | 79.62 | 70.51 | 85.54 | 78.56 | 7.57 |
| 18D | 92.81 | 85.88 | 99.46 | 92.72 | 6.79 |
| 21D | 102.47 | 94.93 | 109.58 | 102.33 | 7.33 |
| 25D | 111.3 | 104.2 | 118.62 | 111.37 | 7.21 |
| 28D | 116.58 | 110.1 | 123.86 | 116.85 | 6.88 |
